# Accuracy of four intraoral scanning devices and the effect of three different illuminance conditions

**DOI:** 10.4317/jced.62602

**Published:** 2025-11-30

**Authors:** Ernesto Edilberto Vilchez Quintana, Gabriel Nima, Luis Andre Mezzomo, Jorge Noriega, Yolanda Natali Raico Gallardo

**Affiliations:** 1 DDS. Master’s candidate, Faculty of Dentistry, Universidad Científica del Sur, Perú; 2 DDS, MS, PhD. Assistant Professor, School of Dentistry, Department of Biomedical Sciences, Ethics, Research and Education, Universidad de los Andes, Chile; 3 DDS, MS, PhD. Clinical Associate Professor, Department of Restorative Dentistry, UIC - University of Illinois at Chicago; 4 DDS, MS. Professor and Coordinator, Specialty in Periodontics and Implantology, Universidad de Ciencias Aplicadas, Perú; 5 DDS, MS, PhD. Professor, Laboratorio de Investigación en Odontología Digital, Faculty of Dentistry, Universidad Científica del Sur, Perú; 6 Assistant Professor, Department of Prosthodontics, School of Dental Medicine, University of Pittsburgh, Pittsburgh, Pennsylvania, USA

## Abstract

The objective of this study was to evaluate in vitro the accuracy of four intraoral scanners and the effect of three different illuminance conditions. A maxillary master cast was digitized with an extraoral scanner to get the reference STL file. The following four intraoral scanners were evaluated: TRIOS 3 (TR), CS 3600 (CS), Helios 600 (HS), and Virtuo Vivo (VV). Three groups were created under different light conditions: 500 lux, 1000 lux, and 6000 lux. Twelve scans were taken for each scanner, resulting in144 STL files in total. The accuracy was calculated with a metrology software. Statistical analysis was obtained by using a generalized linear model (GLM). A Two-Way ANOVA (scanners*light source) and Tukey's post-hoc test (=0.05) were conducted to compare the four scanners among three light conditions. VV showed the best trueness (29.16 ± 11.53 µm) and precision (28.95 ± 11.60 µm) for all illuminance conditions among the tested scanners. Significant differences in accuracy were found in TR when increasing illuminance. No statistical differences in accuracy were found for CS, HS and VV among all three light conditions. VV showed the best accuracy among all light sources and scanner types.

** Key words:**Accuracy, Digital impression, Intraoral scanner, Prosthodontics.

## Introduction

The implementation of technology in dentistry is evolving, and many clinicians nowadays have access to intraoral scanners (IOS) (1). Digital impressions offer a clinically acceptable accuracy, providing restorations with similar or even better marginal adaptation as the conventional methods (2 - 5). Hence, there are many options of IOS systems available on the market, which offer a variety of benefits such as: caries detector, time efficiency, accuracy, ergonomics, artificial intelligence assistance, user-friendly software, patient excitement applications, and patient comfort (1 , 6 - 11). An IOS offers a direct digitization of the patient´s mouth. This device has a tip that captures little digital images of the intraoral environment. These images are converted and processed to 3D image data, by mathematical models and specialized algorithms in the computer (9 , 12). Whatever the image-capturing technology used by the IOS is, the most essential thing that the clinician must know, is that every single dental scanner uses some source of light to scan an object. Therefore, all IOS systems work under optical principles and have their own light source (8). The accuracy of a scanner, according to the International Organization of Standardization (ISO) 5725-1 and DIN 55350-13, is the combination of trueness and precision (13 , 14). Trueness refers to how a scanner generates a digital representation of a dental arch, as similar as possible to reality. Precision refers to how repeatable and consistent, these digital representations are acquired by consecutive scanning under the same conditions (13 - 15). Many factors that influence the accuracy of an IOS have been described in the literature (16 - 24). Indeed, one of the factors that has a significant impact on accuracy, is light condition (25 - 33). A recent systematic review concluded that 1000 lux is recommended when scanning full arches (25). However, for shorter scans, the influence of illumination on the accuracy of the scanning procedure was not clinically relevant (25). The industry has a wide range of options for scanning devices that are becoming increasingly more affordable for clinicians. However, many of these options need more scientific evidence to support their superiority (5). To better benefit from this technology, clinicians should be appropriately informed about the factors that can influence the accuracy of the IOS systems available on the market. This will enable them to choose the best option and make a more accurate decision. Thus, the aim of this study was to compare in vitro the accuracy of four commercially available intraoral scanners and the effect of three illuminance conditions. The null hypotheses were: 1) no significant difference would be found in the accuracy of four intraoral scanners when using the same illuminance condition, and 2) three illuminance conditions would not affect the accuracy of four intraoral scanners.

## Material and Methods

A reference maxillary arch was digitally designed (Exocad, GmbH, Darmstadt, Germany) and printed with dental model resin (Die and Model Tan 2, SprintRay, Los Angeles, USA) in a DLP 3D printer (Pro55S, SprintRay, Los Angeles, USA). The manufacturer's indications for post-processing and curing were followed to obtain the master cast. Subsequently, the master cast was scanned with a laboratory blue light structured scanner (Ineos X5, Dentsply Sirona, Salzburg, Austria). According to the manufacturer, this scanner reports an accuracy of 2.1 ± 2.8 m. After the scanning process, the STL was exported and used as the reference STL file. Four intraoral scanners were evaluated: TRIOS 3 (3Shape, Copenhagen, Denmark), CS3600 (Carestream, Rochester, USA), Helios 600 (Eighteeth, Changzhou, China), and Virtuo Vivo (Dental Wings Inc., Montréal, Canada). The characteristics of the scanners and scanning patterns used in the present study are listed in Table 1.

Table 1

**Figure 1:**
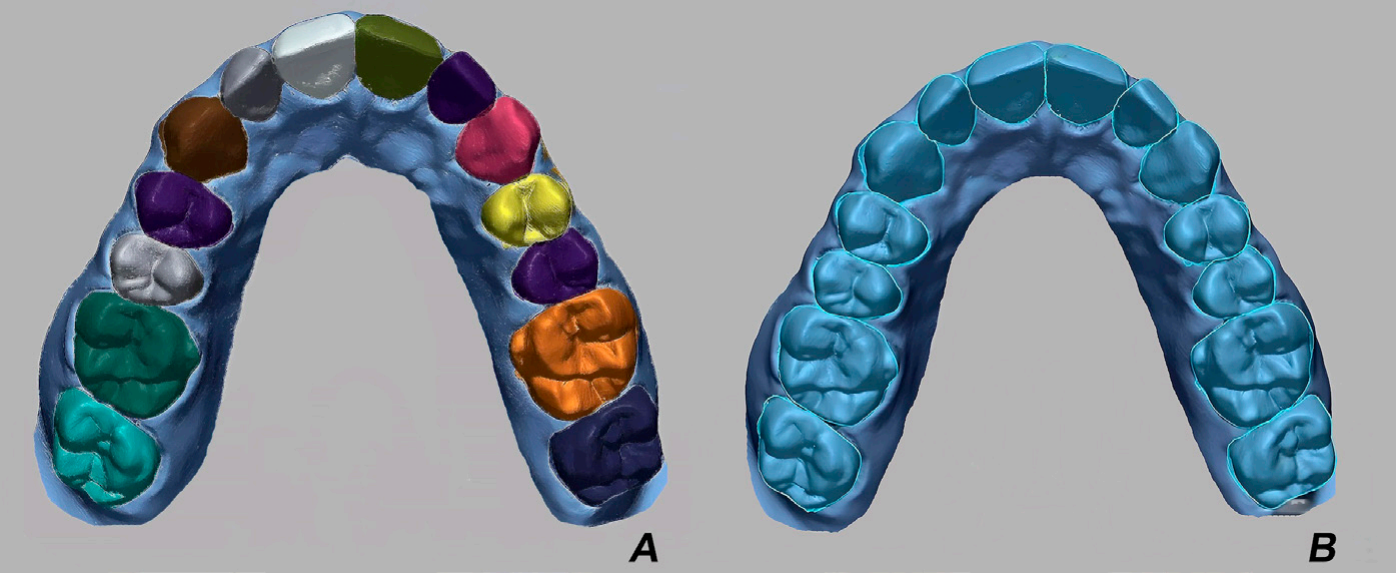
A, Selection of teeth area. B, Best-fit alignment.

The master cast was fixed on an articulator table to avoid an incorrect position or involuntary movements during the scanning process. TR and VV were calibrated before scanning, when environment lighting conditions changed, following the manufacturer´s instructions. On the contrary, CS and HS have a self-calibration system. Each scanner was operated by an expert clinician in each IOS system with an experience greater than two years. The same room temperature (23°C) was used for all the scans following the ISO-12836 specifications based on a previous study (21). To standardize the ambient light condition, all the scanning procedures were performed in the same room. Three illuminance groups were created and the lux for each group was measured by using a lux meter app (LightMeter, Nipakul Buttua, Thailand). In addition, the light condition was measured before the scanning procedure among the three illuminance groups for each scanner tested. The characteristics of the three different illuminance conditions were as follows: a) 500 lux: natural light condition. The ceiling light of the room was turned off, and the windows allowed natural light into the room, which had an illuminance of 500 lux. The scanning process was performed for four days (one day per scanner) at the same daytime. b) 1000 lux: room light condition. The ceiling light of the room was turned on, and the illuminance value of 1000 lux was measured using the lux meter. c) 6000 lux: The ceiling light of the room was turned off, and the LED light of the chair was turned on at a standard 6000 lux value. The position of the LED light was standardized at a 90-degree orientation and at a 60 cm distance to the master cast. Based on a previous study (29), twelve scans were made with the four scanners tested for each illuminance group. Consequently, 144 scans were carried out in total. To avoid dimensional changes, the master cast was stored in a black airtight container without light exposure and humidity. All comparison scans were made within one week following master cast production (34). The STL file of the reference cast and the STL file of the test groups were superimposed and analyzed in a metrology software program by using the "best-fit alignment" algorithm (Geomagic Control X 3.0, Oqton, San Francisco, USA) (Fig. 1).

1

**Figure 2:**
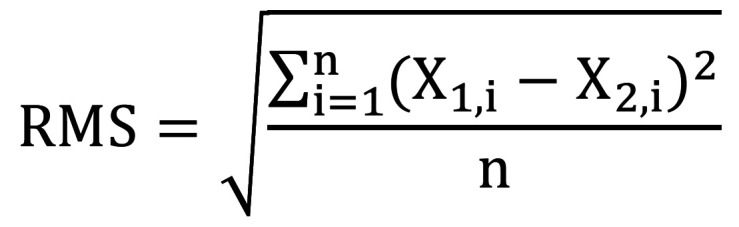
Formula.

The best-fit alignment enabled the evaluation of the two superimposed scans CAD (reference) versus CAM (comparison scan). After the alignment process, a 3D comparison was performed to determine the minimal distance between these two STL files. The 3D discrepancy was assessed using the root mean square (RMS), a measure of the overall magnitude of deviations, calculated with the following formula: (Fig. 2).

2

Where X_(1,i) represents the STL of the design (CAD), X_(2,i) represents the STL of the comparison groups (CAM), and n denotes the total number of measurement points included. Trueness was determined using the average RMS, while precision was calculated based on the standard deviation (SD) of the RMS. Data on trueness and precision underwent tests for normality and homogeneity of variances with Shapiro-Wilk tests (=0.05). The results indicated that the dataset did not exhibit normal distribution. Subsequently, data were analyzed by using a generalized linear model (GLM). A Two-Way ANOVA (scanners x light source) and Tukey's post-hoc test (=0.05) were conducted to compare the four scanners under the influence of three different light sources. All statistical analyses were carried out with SPSS software (v.26) on MacOS 13.5.2.

## Results

**Figure 3:**
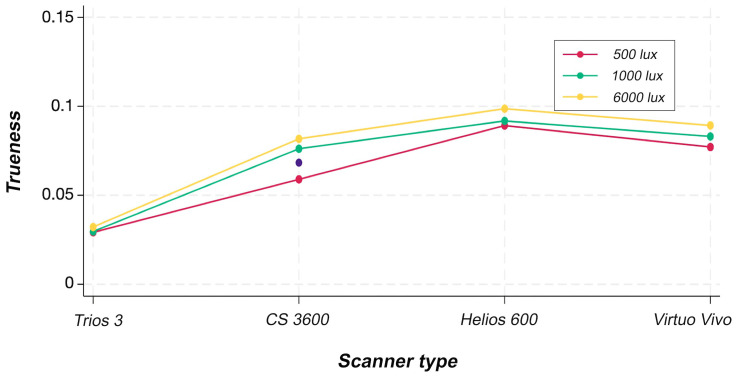
ANOVA plot for trueness.

The results for trueness are summarized in Table 2 and Figure 3.

Table 2

3 4

**Figure 4:**
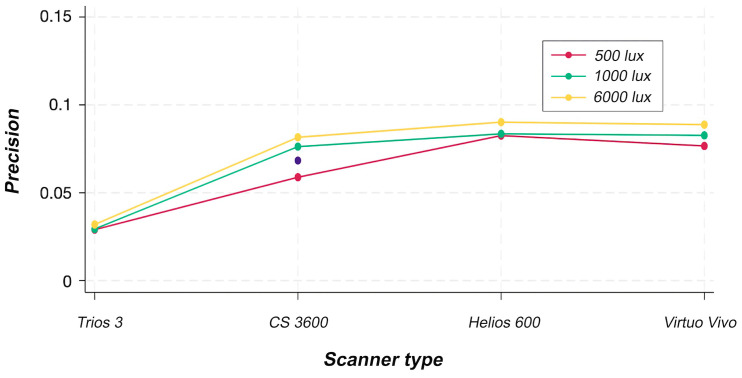
ANOVA plot for precision.

Two-way ANOVA indicated statistically significant effects of the scanner factor (p &lt; 0.001) and of the interaction between scanner and light source (p &lt; 0.001). In contrast, the light source factor showed no statistically significant effect (p = 0.433). Among all devices tested, the VV scanner achieved the highest trueness values across all light source conditions, with statistically significant differences compared with the other scanners. For precision, the results are summarized in Table 3 and Figure 4.

Table 3

5

**Figure 5:**
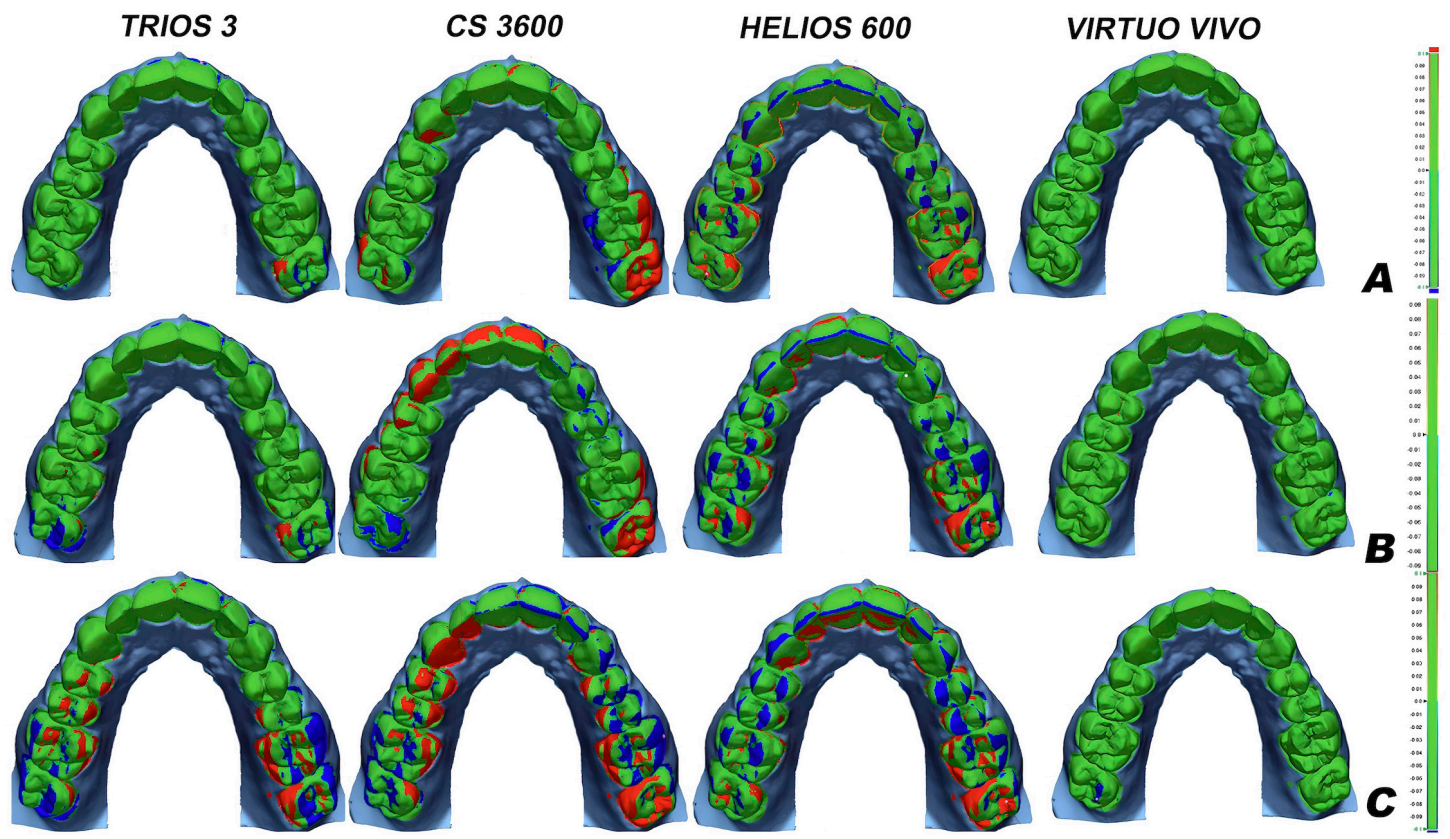
Representative color map of trueness analyses with a metrology software.

Two-way ANOVA indicated statistically significant effects of the scanner factor (p &lt; 0.001) and of the interaction between scanner and light source (p = 0.002). In contrast, the light source factor showed no statistically significant effect (p = 0.410). The precision of the TR scanner was reduced when 6000-lux and 1000-lux light sources were used, whereas the VV, CS, and HS scanners maintained consistent precision across all light source conditions. Figure 5 shows a color map of a representative sample of trueness from each scanner tested under the three illuminance conditions.

Higher deviations were observed in molar regions. This figure displays a color scale where red indicates positive differences &gt;+0.1 mm (an expansion of the real size), and blue indicates negative differences &gt;-0.1 mm (a shrinkage of the actual size).

## Discussion

This study revealed significant differences in the accuracy of four intraoral scanners with the same illuminance source. Therefore, the first null hypothesis was rejected. The second hypothesis was partially rejected because significant differences in trueness values were found for TRIOS 3 (TR) among the three scanning illuminance conditions. However, no significant differences in trueness were found for CS 3600 (CS), Helios 600 (HS) and Virtuo Vivo (VV) among the three scanning illuminance conditions. This study found that scanning accuracy may be influenced by increasing light intensity. Previous studies have also demonstrated that light condition affects the accuracy of an IOS (25 - 33). However, there is no ideal or universal illuminance source for all IOS systems available on the market (26 , 28 - 31). Jivanescu et al. (27) included a single IOS in their study. No significant influence of light condition on scanning accuracy was found. In contrast, Revilla-León et al. (31) reported a decrease in IOS accuracy within 7% to 43% with different light source. The same study found that an illuminance of 1003 lux for TR achieved the lowest absolute error of full-arch scans (31). Although trueness and precision values obtained in this study exhibited a slight influence of illuminance condition, it was not statistically significant in all scanner types. IOS systems have different optical methods to create a 3D image. Therefore, clinicians must be careful when selecting the IOS system and learn about its characteristics and recommendations for better use. The accuracy of a full arch scan in this study ranged in terms of trueness, within 17.63-119.6 µm, and in terms of precision, within 17.35-110.95 µm. Deviations under 100 µm have been reported to be clinically acceptable for complete arch digitalization with an IOS (22). In a previous in vitro study of full arch scans, CS showed a trueness of 61.4 ± 17.3 µm and a precision of 63.2 ± 26.1 µm (28). Moreover, VV reported a mean trueness and precision of 52 µm and 37 µm respectively, for single tooth preparation (17). In the same study, TR reported a mean trueness and precision of 40.5 µm and 11 µm respectively (17). According to the reviewed literature, this appears to be the first study to evaluate and report on the accuracy of Helios 600 scanner. The results of this study showed more errors in molar regions. A possible explanation is that this area has a more complex geometry when compared with anterior teeth (22). Similar results for TR were found in a previous study, with more deviations in molar regions (trueness = 48 µm and precision = 41 µm) (18). Indeed, the literature reported, that as the scan area increases, the accuracy decreases (19 , 24). The explanation for this may be, that when an IOS is capturing the whole arch, more stitching process of the images must be done. Consequently, more errors could be introduced into the entire process of creating the 3D printed cast (19 , 24). For these reasons, a recent study recommends half scans for fabricating tooth-supported crowns instead of complete arch scans (35). Soft tissues may cause inaccuracies or difficulties in the alignment process due to the lack of reference points (11 , 36). Thus, in this study, only the teeth area was selected for the best-fit alignment to improve measurement accuracy (37). These surfaces were occlusal, incisal, vestibular, and palate. Metrology software has been described as a reliable tool for 3D analysis (38). However, previous studies that evaluated the influence of light conditions on the accuracy of IOS systems used several options to perform these analyses. These analyses include distance between two points (28), RMS calculation (29 , 30), average percentile (26 , 27), or distance error metric (31). Hence, the methodology heterogeneity evaluating the 3D accuracy among these studies made it difficult to compare the present results. Moreover, most of these previous studies evaluated only one type of scanner. Studies comparing the accuracy of CS, HS, and VV with different illuminance conditions are lacking. Each intraoral system is composed of hardware and software. Companies are constantly developing hardware's to be more comfortable for the patient and clinician and more affordable with easy clinical access. However, studies have demonstrated that software significantly impacted the accuracy of an IOS (20). For this reason, it is essential to update the software version of the IOS constantly. In this study, multi-scan technology represented by VV showed significantly less distortions (mean trueness: 29.16 ± 11.53 µm; mean precision: 28.95 ± 11.60 µm) even when the hardware has been the same since 2019. The constant software refinements of this scanner may increase its accuracy and performance. To avoid the risk of bias on the results, the scanning process was performed over the same static master cast (with no moisture) by an expert clinician on each scanning system under similar conditions: lightroom, temperature, and scanning path recommended by the manufacturer. However, the results of this in vitro study should be interpreted with caution since only one type of surface was evaluated. Thus, a resin for 3D printing has different optical properties, such as smooth surfaces and regular shapes. Whereas, in the mouth, different surfaces can be found, like restorative materials, scan bodies, and tissue density, among others. Moreover, as in an in vitro study, it is impossible to simulate saliva, blood, breathing, patient´s and operator´s movement, or other characteristics of the intraoral cavity environment. Another limitation of this study is that the IOS systems evaluated are not the latest models available on the market. However, since the investment to acquire an IOS is high, many clinicians are still using these models. The software of these IOS systems is continually being updated, making them usable even though they are old models. Further clinical investigations are advised to compare the impact of different light sources on the accuracy of IOS systems available on the market. Future studies should have a consensus on the methodology for 3D analysis, such as standardized protocols for the alignment process and distance error calculation.

## Conclusions

Based on the findings of this in vitro study, the following conclusions can be drawn: -The light condition may affect the scanning accuracy depending on the IOS selected. Although a decrease in the accuracy of the four scanners tested under different light conditions was observed, it was not statistically significant for all of them. -Virtuo Vivo had the best trueness values among all scanner types and light sources. -CS 3600 had the lowest precision values among all scanner types and light sources.

## Figures and Tables

**Table 1:** Table Characteristics of intraoral scanners.

IOS System	Company	Imaging technology	Image Type	Light source	Scanning path recommended by manufacturer	Software version
TRIOS 3	3Shape, Denmark	Confocal microscopy	Photographic	White	Start on the right molar from the occlusal surface, followed by the buccal and palatal	1.4.7.3
CS3600	Carestream, USA	Active triangulation technique	Videographic	Purple	Start on the right molar from the occlusal surface, followed by the buccal and palatal	1.0.9.7
Virtuo Vivo	Dental Wings, Canada	Laser-Multiscan	Videographic	Blue	Start scanning the occlusal face on the right molar- when you reach the canine, switch to a rocking motion- finish scanning the occlusal surface- rotate to lingual 45° and scan the full arch- rotate to buccal 45°and scan the full arch	3.10
Helios 600	Eighteeth, China	Not available	Not available	White-Blue	Start on the right from de occlusal surface, slowly wiggle the scanner when passing the centrals, then lingual and bucal surfaces.	1.1.4.4

1

**Table 2:** Table Mean values ± standard deviations in µm for trueness of the scanners under different light sources.

	Trios 3	Carestream	Helios 600	Virtuo Vivo
500 Lux	60.31 ± 14.34 Ab	89.16 ± 17.93 Ac	77.11 ± 4.80 Ac	29.16 ± 11.53 Aa
1000 Lux	75.51 ± 17.16 Bb	91.75 ± 14.85 Ac	82.83 ± 8.39 Abc	29.64 ± 3.15 Aa
6000 Lux	81.69 ± 26.50 Bb	98.62 ± 20.98 Ac	88.90 ± 8.88 Abc	32.18 ± 3.76 Aa

Standard deviations followed by different letters indicate significant differences. Uppercase letters compare light sources within the same scanner, whereas lowercase letters compare scanners within the same light source, according to the two-way ANOVA and Tukey’s post-hoc test (p< 0.05). Lower values indicate better trueness.

**Table 3:** Table Mean values ± standard deviations in µm for precision of the scanners under different light sources.

	Trios 3	Carestream	Helios 600	Virtuo Vivo
500 Lux	60.36 ± 14.68 Ab	82.50 ± 19.11 Ac	76.61 ± 5.02 Ac	28.95 ± 11.60 Aa
1000 Lux	75.45 ± 17.18 Bb	83.52 ± 14.65 Ab	82.48 ± 8.45 Ab	29.37 ± 2.90 Aa
6000 Lux	81.53 ± 26.43 Bb	90.16 ± 20.79 Ab	88.36 ± 9.03 Ab	31.91 ± 3.66 Aa

Standard deviations followed by different letters indicate significant differences. Uppercase letters compare light sources within the same scanner, whereas lowercase letters compare scanners within the same light source, according to the two-way ANOVA and Tukey’s post-hoc test (p< 0.05). Lower values indicate better precision.

## Data Availability

The datasets used and/or analyzed during the current study are available from the corresponding author.
